# Are students performing the same in E-learning and In-person education? An introspective look at learning environments from an Iranian medical school standpoint

**DOI:** 10.1186/s12909-023-04159-7

**Published:** 2023-04-04

**Authors:** Haniye Mastour, Ali Emadzadeh, Omid Hamidi Haji Abadi, Shabnam Niroumand

**Affiliations:** 1 Department of Medical Education, Faculty of Medicine, Mashhad University of Medical Sciences, Mashhad, Iran; 2 Department of Medical Education, School of Medicine, Mashhad University of Medical Sciences, Mashhad, Iran; 3 Mashhad University of Medical Sciences, Mashhad, Iran; 4 Department of Community Medicine, Faculty of Medicine, Mashhad University of Medical Sciences, Mashhad, Iran

**Keywords:** COVID-19, Pandemic, E-Learning, In-Person education, Medical education, Undergraduate student, Learning environment

## Abstract

**Introduction:**

This study investigated medical students’ intended learning outcomes based on e-learning and in-person education.

**Methods:**

In this cross-sectional comparative analytical study, a group of 126 undergraduate medical students’ intended learning outcomes under two different teaching methods, including e-learning and in-person, were repeatedly measured based on the census sampling method. Participants were in the preclinical curriculum phase (physiopathology) at Mashhad University of Medical Sciences (MUMS), Iran. Due to expert panel opinion, the same medical teachers and similar difficulty of lessons were considered in two investigated academic semesters. In addition, difficulty and discrimination indexes of formative and summative assessments were controlled for two study groups. The students’ learning outcome index was the knowledge test scores participants received in the relevant lessons of the General Medicine (GM) curriculum preclinical courses.

**Results:**

The findings indicated that students learning outcomes were significantly higher during e-learning than in in-person education for all examined variables (*P* < 0.05). Moreover, the difference between students’ Grade Point Average (GPA) categories among the two groups was significant (*P* = 0.022). Students with a GPA of less than 14 experienced higher increments in their average scores after the e-learning compared to in-person education. Compared to face-to-face courses, improvements in pharmacology, theoretical semiology, and pathology scores after e-learning courses were statistically significant (P < 0.001). The differences in mean scores related to practical pathology and semiology in the two approaches were not statistically significant, P = 0.624 and P = 0.149, respectively. Furthermore, the overall students’ average scores increased significantly during e-learning versus in-person education (P < 0.001).

**Conclusion:**

We concluded that e-learning could be appreciated as a successful method of medical education and can be used as an alternative educational method. However, considering the importance of practical or clinical courses in medical education, further research about the efficacy of the e-learning approach is highly recommended.

## Introduction

The pandemic of COVID-19 infection has interrupted the educational programs in different universities, especially in medical schools all over the world [1]. Most medical schools were suspended from their regular face-to-face courses to ensure the safety of their students and faculties. After that, e-learning was added as one of the core teaching methods throughout the COVID-19 pandemic [2, 3]. This technology refers to the web-based software for distributing, tracking, and managing curriculum-based internet programs between students and faculty [4]. E-learning might be included structures like whiteboards, chat rooms, polls, quizzes, discussion forums, and surveys to be allowed the lecturers and students to communicate online and share course content side by side [5]. E-learning provides facilities for many training aspirations, including independent learning, self-directed learning, special time-independent learning, collaborative training, and real-time feedback [6]. Therefore, students increasingly use e-learning as a complementary educational method. Different e-learning methods are available for medical education, such as online learning platforms, e-books (electronic versions of standard textbooks), e-journals, online question banks, medical websites, and mobile phone apps [7]. Students declared that substituting electronic books and online articles during teaching could increase the active attitude toward learning [8, 9]. However, the success of the e-learning teaching method is influenced by many variables, consisting of access to suitable practice, the course content, and demographic criteria for students. In addition, advantages having improved convenience, more accessible resources irrespective of the location and time, and reduced costs must be deliberated [10–14]. Although conventional learning methods such as the in-person approach have been approved for many years, e-learning courses have been recognized as efficient learning modalities in different educational and governmental studies [8, 15]. In low- and middle-income countries (LMICs), e-learning for medical education may alleviate the burden of severe health worker shortages and deliver affordable access to high-quality medical education [16]. Concurrent with stressing recent advancements in educational technology, researchers believe that the learning environment is one of the causes of satisfying the learners’ diverse requirements. So, the growing demand for the extension service for e-courses has created issues that need comprehensive consideration [17].

Consequently, investigating students’ learning in different teaching methods seems necessary. For many years, it was believed that face-to-face training has a more significant impact on students’ performance than e-learning and distance methods. Until the COVID-19 pandemic, the virtual learning environment was mainly used as a teaching aid. Therefore, pursuing this particular learning method’s effectiveness in the students’ learning outcomes was impossible. During the coronavirus pandemic, due to the complete mandatory shift from face-to-face education to e-learning, it was an opportunity to evaluate students’ performance in virtual learning environments. This study aims to compare the students’ learning outcomes between in-person and e-learning methods.

## Methods

In this analytical cross-sectional study, a group of 126 undergraduate medical students’ intended learning outcomes under two different teaching methods, including e-learning and in-person, were repeatedly measured based on the census sampling method. Participants were in the preclinical curriculum phase (physiopathology) of the GM (General Medicine) at MUMS (Mashhad University of Medical Sciences), Iran. The same set of students was considered for both teaching modalities. The students’ learning outcomes were assessed due to the knowledge test scores they received in the relevant lessons of the GM curriculum preclinical courses from September 2020 to August 2021. The background characteristics were mainly students’ gender, age, residency status (native and non-native), type of university admission (free tuition, paying tuition, and international students), and GPA. To ensure the similarity and difficulty of lessons, the same courses in two consecutive semesters were evaluated. These courses, including pharmacology, pathology, and semiology, were taught in two successive semesters due to the large volume of educational materials in the GM curriculum. Students attended regular face-to-face classes for mentioned courses which consisted of pharmacology I, theoretical and practical pathology I, and theoretical and practical semiology I from September 2020 to February 2021. In the in-person training, the educators taught lessons using lectures and interactive presentations; at this point, questioning was allowed. After that, the students who successfully passed these courses entered the new semester and were educated on the intended courses under the e-learning approach and attended pharmacology II, theoretical and practical pathology II, and theoretical and practical semiology II from February 2021 to August 2021. Participants who failed in face-to-face training courses were excluded from further analysis.

Methods of providing e-content for e-learning, modes of interaction, and many other factors in online learning are classified into synchronous and asynchronous. Synchronous e-learning is live, real-time, facilitating instruction and learning-oriented interaction [17]. Asynchronous online learning is a time-delayed interaction that the communication of both instruction and learning is not occurring simultaneously [18]. The e-learning content was delivered to students in several interactive multimedia formats (slide-based lectures, standalone training videos, embedded videos, webinar or live training playback, podcasts, dialogue simulations, animations, and interactive videos) with formative and summative assessment modules at the end of each Learning Object (LO). The LOs were designed and created in collaboration with instructional designers and electronic content developers under the supervision of an expert panel, including relevant faculty members. During the e-learning, students received the courses via the Learning Management System of MUMS (NAVID®, https://mumsnavid.vums.ac.ir) for asynchronous online learning and Adobe Connect and Skyroom for synchronous e-learning. All theoretical and practical courses assessed in this study were delivered to students similarly based on the curriculum.

All methods were carried out following relevant guidelines and regulations. The study protocol was aligned with the ethics committee (the approval date was 2020-08-18 with IR.MUMS.MEDICAL.REC.1399.298 reference code). All registered students voluntarily agreed to participate in the study. Informed consent was obtained from all subjects. Also, to maintain confidentiality, all data were analyzed anonymously and based on an identification code.

**Fig. 1:**
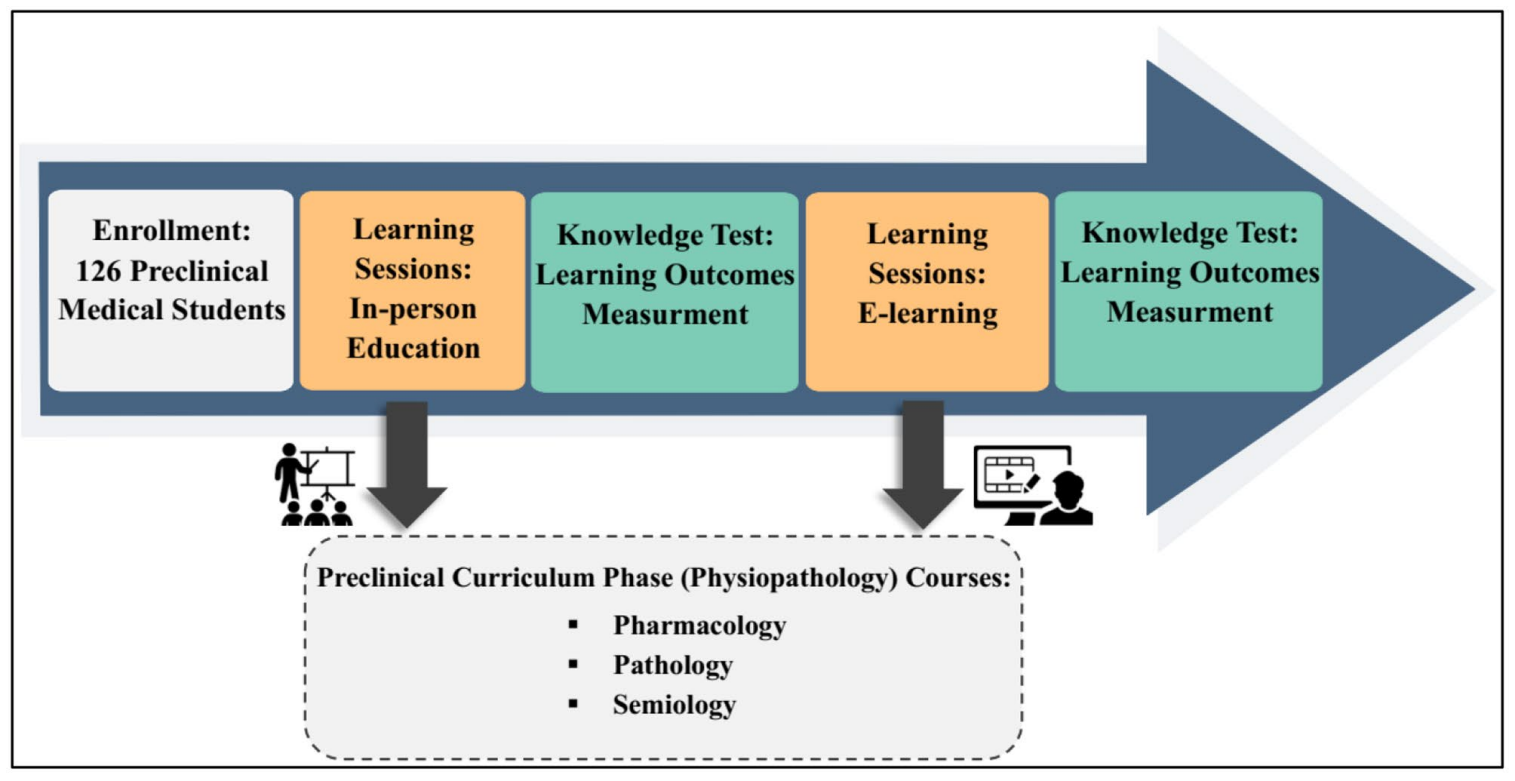
The steps of this study

The data collection tools for students’ intended learning outcomes were knowledge tests in both groups. The test questions were designed and developed based on the content of the courses taught and consisted of 20 Multiple Choice Questions (MCQ) with one correct option out of four for each lesson. The maximum score of the tests was 20 points, and there were no partial or subtracted points. Due to the expert panel’s opinion, the same medical teachers and lessons that were similar in difficulty were considered in two investigated academic semesters. However, these courses were the prerequisite to each other and were related vertically during the educational curriculum. Nine medical faculty members approved the validity and reliability of the questions for both semesters, and Cronbach’s alpha was in the range of 0.79 to 0.86. Difficulty and discrimination indexes of formative and summative assessments were controlled for two study groups. In our university, the electronic examination system has the possibility of various reports, including the difficulty and discrimination indexes of questions. Therefore, the system report was used to check the questions extracted from the question bank (https://qsanjesh.mums.ac.ir), and the questions in both groups were homogeneous in terms of the mentioned criteria. The steps of the current study are demonstrated in Fig. 1.

In this study, the students of the in-person education participated in the onsite proctored exams. Yet, for individuals who attended the e-learning, due to the limits of COVID-19, we had to hold the formative and summative assessments online in the MUMS central examination system (https://esanjesh.mums.ac.ir). This evaluation method has some challenges, including the necessity of matching with technology, infrastructural barriers, especially in our country as a developing one, and the probability of cheating in online exams. To ensure the privacy of the online tests, we attempted to detect cheating during the exam by verifying the student’s identity through continuous authentication. For added security, the exam-taker was required to answer a short series of security questions based on background student information extracted from the MUMS educational system. In addition, we supervised remote exam-takers by watching live online videos to identify suspicious examinee behaviors or items in their immediate environments, such as repeatedly glancing away from the screen. Since the effect of cheating in online exams was an important confounder in this study, we applied other ways to reduce it, including randomizing examination questions and supplemental oral tests using online video calls.

Then the students’ knowledge test scores in the lessons of courses mentioned above (pharmacology, pathology, and semiology) were collected. The participants’ background information was extracted from the university educational system to compare their intended learning outcomes in e-learning and in-person learning environments. We analyzed data under the statistical package for the social sciences version by SPSS (SPSS Inc., version 20, Chicago, Illinois, USA). The variables with a continuous normal distribution were defined in the text by the Mean ± Standard Deviation. Also, the qualitative variables were determined by frequency and percentages in their related groups. The Shapiro-Wilk test was used to define the distribution of variables. The comparisons between two study groups with normal distribution have been performed under Student’s T-Test independent samples. Analysis of Variance by ANOVA Test was committed to comparing the difference between two groups’ quantitative categorical variables. Furthermore, paired T-Test was performed to analyze the difference between e-learning and in-person intended learning outcomes. A *P*-value less than 0.05 was considered statistically significant.

## Results

In this cross-sectional study, 126 undergraduate medical students participated. The participants’ background characteristics showed that most students were male (52%) and were admitted for free tuition (83%). Related features showed that most participants had a GPA between 14 and 17 (65%). Furthermore, most of the students were non-native (53%) in our study and had less than 24 years old (78%) (Table 1).

**Table 1:** Background characteristics of medical students

Variables		Frequency (Percent)
Gender	Female	61 (48.4)
	Male	65 (51.6)
Age (Years)	≤ 21	49 (38.9)
	= 24	49 (38.9)
	≥ 25	28 (22.2)
Residency Status	Native	59 (46.8)
	Non-native	67 (53.2)
Type of Admission	Free Tuition	104 (82.5)
	Paying Tuition	17 (13.5)
	International	5 (4)
Grade Point Average (GPA)	< 14	16 (12.7)
	14–17	82 (65.1)
	> 17	28 (22.2)

The Comparison of e-learning and in-person methods in medical students’ learning outcomes based on background characteristics is presented in Table 2. The findings indicated that students learning outcomes were significantly higher during e-learning than in in-person education for all examined variables (*P* < 0.05). The relation between teaching method and students’ gender was insignificant (*P* = 0.205) as male and female students had approximately an equal increment of average scores during e-learning compared to in-person education. There wasn’t any statistically significant difference between students’ types of admission (*P* = 0.533) and their residency status (*P* = 0.085) with intended learning outcomes. The students with different age categories had similar average scores in both teaching methods (*P* = 0.546). Moreover, the difference between students’ GPA categories among the two groups was significant (*P* = 0.022). Students with a GPA of less than 14 experienced higher increments in their average scores after the e-learning compared to in-person education.

**Table 2:** Comparison of E-learning and In-person methods in medical students’ learning outcomes based on background characteristics

Variables		Learning Environment Mean ± Standard Deviation			*P*-value	T^**^	df
		In-person	E-learning	Difference			
**Gender**	Female	14.9 ± 1.8	15.8 ± 1.6	1 ± 1.6	< 0.001	-4.7	60
	Male	15 ± 1.5	15.7 ± 1.5	0.6 ± 1.2	< 0.001	-4.2	64
	*P*-value	0.53	0.606	0.205			
	T^*^ df	-0.6 124	0.5 124	1.3 124			
**Age (Years)**	≤ 21	15.3 ± 1.6	16 ± 1.7	0.6 ± 1.2	< 0.001	-3.8	48
	22–24	15 ± 1.4	15.9 ± 1.4	0.9 ± 1.4	< 0.001	-4.9	48
	≥ 25	14.6 ± 1.6	15.3 ± 1.1	0.7 ± 1.7	0.047	-2.1	24
	*P*-value	0.117	0.132	0.546			
	F	2.2	2.1	0.6			
	df	2, 120	2, 120	2, 120			
**Residency Status**	Native	14.8 ± 1.5	15.8 ± 1.3	1 ± 1.3	< 0.001	-5.7	58
	Non-native	15.2 ± 1.6	15.8 ± 1.6	0.6 ± 1.4	= 0.001	-3.4	63
	*P*-value	0.129	0.992	0.085			
	T^*^	-1.5	-0.0	1.7			
	df	121	121	121			
**Type of Admission**	Free Tuition	15.1 ± 1.6	15.8 ± 1.5	0.7 ± 1.4	< 0.001	-5.2	103
	Paying Tuition	14.4 ± 1.3	15.3 ± 1.1	1 ± 1.2	0.009	-3.1	13
	International	15.2 ± 0.6	16.5 ± 1	1.3 ± 0.4	0.002	-6.8	4
	*P*-value	0.247	0.279	0.533			
	F	1.4	1.3	0.6			
	df	2, 120	2, 120	2, 120			
**Grade Point Average (GPA)**	< 14	13.1 ± 1	14.7 ± 1.1	1.6 ± 1.4	< 0.001	-4.6	15
	14–17	14.8 ± 1.2	15.5 ± 1.3	0.7 ± 1.4	< 0.001	-4.7	78
	> 17	16.8 ± 0.9	17.2 ± 1.1	0.4 ± 1.1	0.043	-2.0	27
	*P*-value	< 0.001*	< 0.001*	0.022			
	F	62.1	26.4	4			
	df	2, 120	2, 120	2, 120			

T^*^: Independent Sample T-Test; T^**^: Paired T-Test; F: Analysis of Variance

- Tukey’s honestly significant difference (HSD) post hoc test showed statistically significant diffrences between every two groups based on grade point average.

The Comparison of e-learning and in-person methods in participants’ performance based on different lessons of preclinical medical education is presented in Table 3. Compared to face-to-face courses, improvements in pharmacology, theoretical semiology, and pathology scores after e-learning courses were statistically significant (*P* < 0.001). The differences in mean scores related to practical pathology and semiology in the two approaches were not statistically significant, *P* = 0.624 and *P* = 0.149, respectively. Furthermore, the overall students’ average scores increased significantly during e-learning versus in-person education (*P* < 0.001).

**Table 3:** Comparison of E-learning and In-person methods in participants’ performance based on different lessons of preclinical medical education

Variables	Learning Environment Mean ± Standard Deviation			*P*-value	Test
	In-person	E-learning	Difference		
**Pharmacology**	14.4 ± 2.1	15.6 ± 1.7	1.2 ± 1.8	< 0.001	T= -7.6
**Practical Semiology**	16.6 ± 1.6	16.9 ± 2.4	0.4 ± 2.5	0.149	Z=-1.4
**Theoretical Semiology**	14.7 ± 2.1	15.9 ± 2.1	1.2 ± 2.2	< 0.001	T= -6.8
**Practical Pathology**	16 ± 2.6	15.9 ± 2.3	-0.1 ± 3.2	0.624	Z= -0.5
**Theoretical Pathology**	13.4 ± 2.1	15.6 ± 1.9	2.2 ± 2	< 0.001	T= -12
**Average of Knowledge Test Scores**	15 ± 1.6	15.8 ± 1.5	0.8 ± 1.4	< 0.001	T= -6.3

T: Paired T-Test; Z: Wilcoxon Test

## Discussion

In this study, we compared the intended learning outcomes of undergraduate medical students based on e-learning and in-person methods during the COVID-19 pandemic. It was revealed better learning outcomes based on students’ knowledge test scores attributed to e-learning versus face-to-face education. Besides, some significant differences were found in the two mentioned methods due to background characteristics, including students’ gender, age, residency status, type of admission, and GPA. Previous research showed that simple access to learning subjects and the ability to select the place and the study time is considered the main benefit of e-learning compared to in-person education [19], which was too important during the COVID-19 pandemic. The role of e-learning in reducing accommodation and transportation expenses is a significant factor [20, 21]. The studies emphasize that e-learning enables educational subjects to be delivered to students fast, effectively, updated, and standardized [19, 22]. However, it is too difficult for the students as well as the teachers to conduct/attend classes online, due to which a lot of problems are being faced by both students as well as teachers like student assessment, proper class attendance, internet connectivity issue, and vice versa [23].

In this study, increasing the average scores of medical students indicated that e-learning enables them to improve their knowledge more than in-person education. So, the present study demonstrated that e-learning is an appreciated teaching method for medical students. Schrader (2015) showed that e-learning is 6% more effective than in-person training in providing knowledge. However, students’ learning was reported to be identical both ways, and students were satisfied with both methods [24]. Pallavi et al. (2022) declared that more than 50% of the respondents feel online learning is as effective as conventional classroom learning [25]. In an e-learning environment that emphasizes learner-centered activity and system interactivity, remote learners can outperform traditional classroom students [26]. However, Jiang et al. (2023) obtained that the e-learning behavior of undergraduate students needs to be improved [27]. Previous research supported that enhancing students’ knowledge of e-learning might increase secondary outcomes such as their self-discipline degree [28].

Further, the authors found the differences in mean scores related to practical pathology and semiology in the two approaches were not statistically significant. We considered it more challenging to understand practical courses than theoretical ones in the e-learning method versus in-person education. Since e-learning was less effective in increasing medical students’ achievement for practical semiology and pathology, concentrating on improving the average scores of practical courses besides theoretical lessons is critical. Garland (2010) explored e-learning versus classroom instruction in a Dental Hygiene Program. Findings demonstrated little difference between the two methods [29]. Several studies might support this finding, revealing that indirect e-learning could delay conventional direct learning in many aspects [30]. In this regard, this was similar to recently published investigations that assessed students’ understanding of online classes during the pandemic of COVID-19 [31–33]. This point might be due to the lack of interaction between lecturers and students in e-learning compared to face-to-face education, specifically for practical courses. A previous study considered this point as one of the disadvantages of e-learning compared to a face-to-face learning environment [19]. However, E-learning activities can turn rather dull online experiences into entertaining, interactive, meaningful, and valuable learning experiences for students [34]. Interactive learning environments respond dynamically to learners’ actions and are associated with active, learner-engaged processing of learning materials. Such settings are expected to promote deep cognitive processes and actively construct new knowledge [35]. Previous investigations declared that weak interaction between students and organizers and the lack of transparency regarding the learning goal could prevent the e-learning method’s education process compared to the face-to-face way [36, 37]. Bock et al. (2021) performed a randomized study to compare the effectiveness of face-to-face, blended, and e-learning teaching in local anaesthesia by assessing students’ knowledge gain and performance of practical skills. Their study indicated that blended learning improves the learning outcome for theoretical knowledge in teaching local anaesthesia more than either face-to-face learning or e-learning alone. For acquiring practical skills, their study showed that blended learning was as effective as other teaching methods [38]. Barteit et al. (2020) systematically reviewed e-learning interventions for medical education in low- and middle-income countries (LMICs). According to their findings, most studies self-concluded that they had an effective e-learning intervention, thus indicating the potential benefits of e-learning for LMICs [16]. Further, studies have shown that e-learning modalities are used widely by students outside of their formal curricula and by health professionals for continuing professional education, indicating that students and professionals appreciate and take advantage of e-learning [38]. Individual learners take advantage of self-paced learning environments in which they control their learning pace, information flow, selection of learning activities, and time management [17]. Finally, Frehywot et al. (2013) said, “E-learning in medical education is a means to an end, rather than the end in itself.“ It seems utilizing e-learning can result in more significant educational opportunities for students while enhancing faculty effectiveness and efficiency. [39].

## Limitations of the study

The study has some limitations. Student assessment is one of the most challenging aspects of virtual education since academic integrity could be contravened due to various cheating behaviors in online examinations [40]. Although we tried cheating detection before and during the exams, this was one of the critical limitations of our study because cheating methodologies, types, and facilitators are widespread. Another limitation is that only short-term retention was assessed in our study. The assessment of students’ retention would be essential to determine future studies. Furthermore, studies on the existing literature, evidence, and a variety of e-learning examples could move beyond the determination of an appropriate blend of different instructional strategies, including e-learning, face-to-face/in-person instruction, and performance-based skill practices.

## Conclusion

The present study’s findings could declare that e-learning alone might not have the necessary efficiency in practical and clinical medical education and should be applied alongside the in-person method as a complementary learning opportunity. Consequently, although e-learning may represent an optimal solution to maintain learning processes in exceptional emergencies such as the COVID-19 pandemic, in-person education is more effective for acquiring practical skills than other virtual learning environments.

## Data Availability

All tables and the study results were provided based on the study’s raw data, which is available from the corresponding author. She will send the SPSS file upon your request.
